# Occurrence of second primary malignancies in patients with neuroendocrine tumors of the digestive tract: A case report

**DOI:** 10.1097/MD.0000000000016508

**Published:** 2019-07-19

**Authors:** Angelo Pirozzi, Ferdinando Riccardi, Grazia Arpino, Carmela Mocerino, Severo Campione, Carlo Molino, Giacomo Cartenì

**Affiliations:** a Department of Clinical Medicine and Surgery, University of Naples Federico II; b Department of Medical Oncology, Azienda Ospedaliero-Universitaria; c Department of Pathology; d Azienda Ospedaliero-Universitaria, Naples, Italy.

**Keywords:** colon cancer, DLBCL, multiple neoplasia, neuroendocrine tumor, primary neoplasms, secondary

## Abstract

**Rationale:**

There is an association between the presence of neuroendocrine neoplasms and incremented risk to develop second primary malignancies. This risk is estimated to be 17%. The most common secondary neoplasms were found in the Gastrointestinal and Genitourinary tracts.

**Patient concerns:**

A 74-year-old Caucasian patient with melaena came to our observation in June 2015. The Esophago-gastro-duodenoscopy exam found a polypoid formation in the duodenal bulb. Histopathological examination showed a well-differentiated neuroendocrine neoplasm (G1).

**Diagnosis:**

During the follow up for the neuroendocrine neoplasm, a CT scan was performed in August 2016 describing infiltration of the right renal sinus and the third proximal ureter segment with heterogeneous enhancement of vascular structure. An US-guided biopsy was conclusive for a Diffuse Large B Cell Lymphoma. In October 2016, a colonoscopy showed a neoplastic lesion at 20 cm from the anal orifice. The Histology exam was positive for an adenocarcinoma with a desmoplastic stroma infiltration.

**Interventions:**

In November 2016, the patient underwent a left hemicolectomy: the pathologic staging described a G2 adenocarcinoma pT3N1b. In May 2018, the Octreotide scan was negative. In the same month, the patient started a treatment based on 6 cycles of Rituximab, Oxaliplatin, and Capecitabine due to the persistence of lymphomatous disease and hepatic metastases. In July 2018, other 3 cycles of the same treatment were scheduled.

**Outcomes:**

In January 2019, due to an increase in liver metastases’ size, it was decided to start a new regimen for the colon cancer with FOLFIRI+Cetuximab. The patient is still in treatment with this regimen in April 2019.

**Lessons:**

The risk of a second primary tumor is increased among patients older than 70. Therefore, it is necessary to follow them using total body CT scan and endoscopic techniques of gastrointestinal and genitourinary tracts, not only for the evaluation of the neuroendocrine tumor but also for the higher risk to develop other neoplastic diseases.

## 1 Introduction

Neuroendocrine tumors (NETs) are rare neoplasms that arise from neuroendocrine diffuse system so they can potentially be found in every part of human body. Two-thirds of the cases diagnosed are gastroenteropancreatic neuroendocrine tumors (GEP-NETs).^[1]^ Every year 2500 to 2700 cases of NETs are recorded in Italy. Their frequency is increasing due to the improvements in diagnostic procedures in the last years and a more detailed clinical and histopathological knowledge. The most common primary sites are gastro-entero-pancreatic tract (60%–70%), respiratory system (20%), skin, thyroid, parathyroid, and adrenal glands. No significant risk factor was identified except for age: elderly people have a higher risk to develop NETs. The risk is increased also in patients who have a positive family history for some hereditary diseases such as multiple endocrine neoplasia (MEN)1 (correlated to pituitary adenomas, pancreatic endocrine cancers, and hyperparathyroidism), MEN2A (associated with medullary thyroid cancer, pheochromocytoma, and hyperparathyroidism), MEN2B (related to medullary thyroid cancer, pheochromocytoma, hyperparathyroidism, Marfanoid habitus, and intestinal ganglioneuromatosis).^[2]^ Negative prognostic factors for NETs are the primary site (e.g., pancreatic NETs have a worse prognosis than intestinal NETs), stage, morphology and proliferative activity expressed as number of mitotic divisions or proliferative index (ki67).^[3–6]^ Considering ki67 value, it is possible to distinguish patients in 3 different categories of risk. In fact, it is recognized as the most important prognostic factor.^[7]^ Its value is related to the OS and disease progression in advanced NETs^[8,9]^ and patients who undertook surgery for a pancreatic NET.^[10]^ Other significant prognostic factors are the expression of somatostatin receptors, which indicates a more favorable evolution, age, and rapidity of progression. From a clinical point of view, GEP NENs are commonly divided in functional and non-functional tumors based on the capacity to produce and release hormones. Functional NETs cause clinical symptoms related to the type of hormone release.

## 2 Case presentation

**Figure 1:**
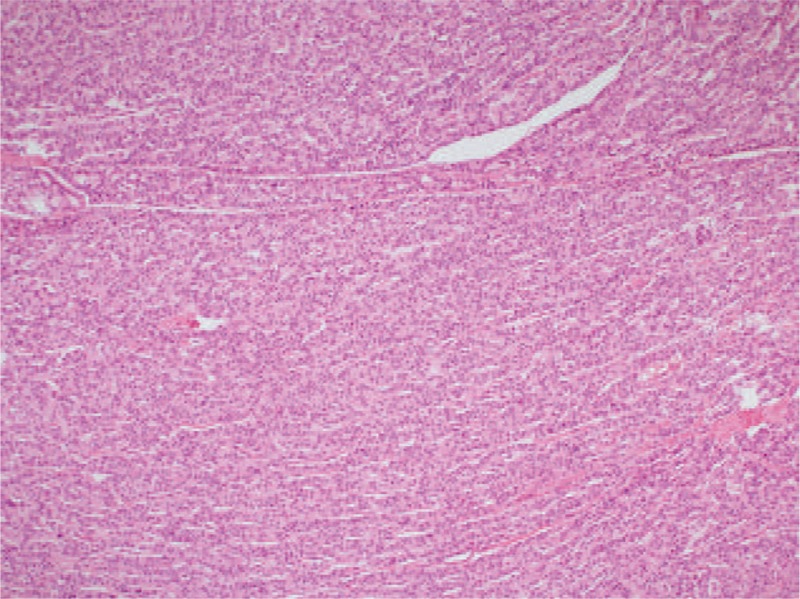
Low grade (G1) neuroendocrine tumor with preserved and well differentiated structure,(H&E × 100).

**Figure 2:**
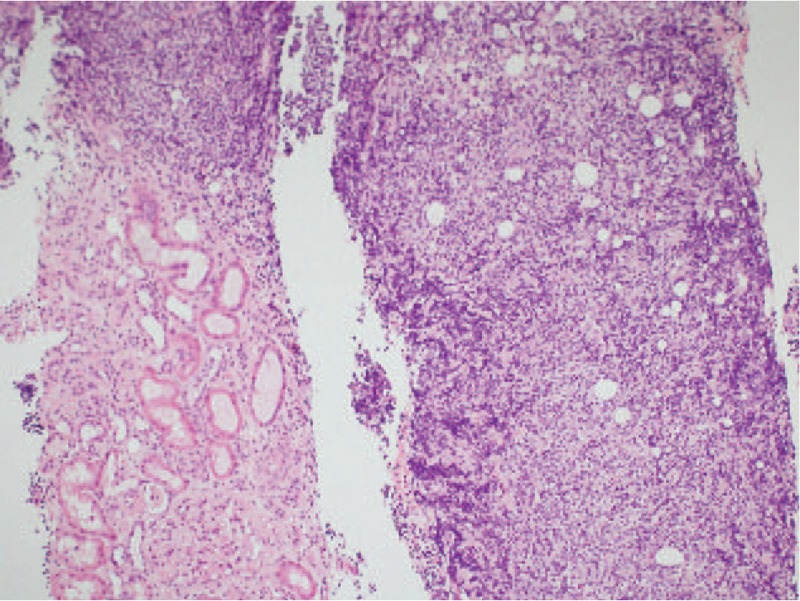
Differences between normal kidney tissue (left) and Lymphoma tissue (right) which has replaced and altered the original structure (H&E × 100).

**Figure 3:**
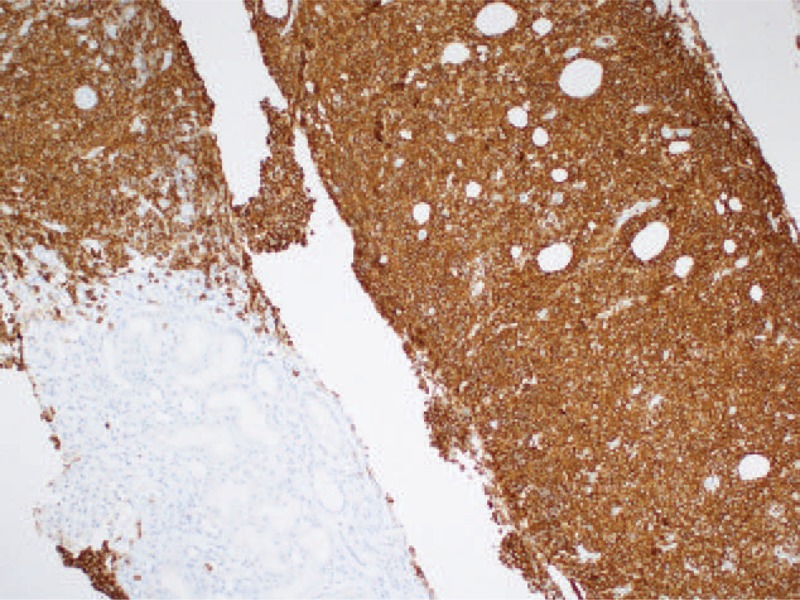
Positive IHC stain for CD20 showing sheet-like lymphocytes on the right (×100).

**Figure 4:**
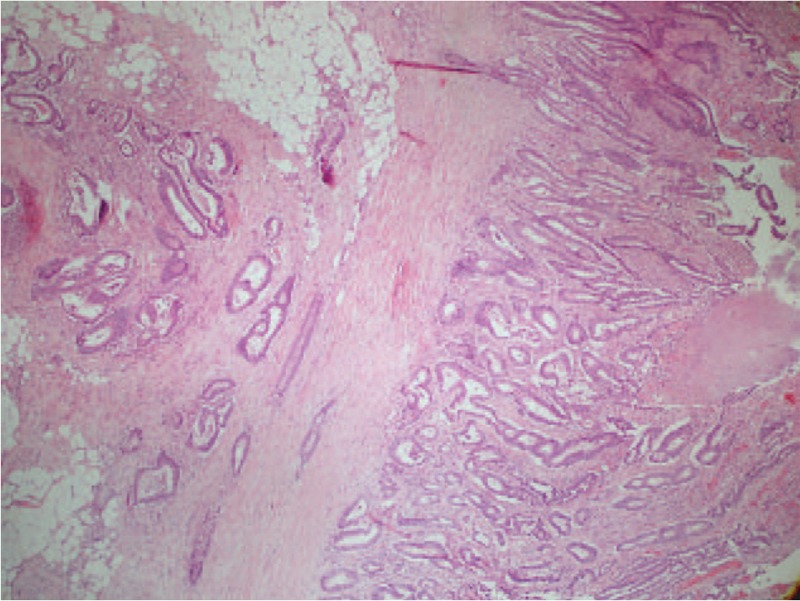
Colorectal Cancer sample: on the left, disorganized glands invading the muscular tissue and underlying layers (H&E × 100).

Our patient was a 74-year old Caucasian man affected by chronic obstructive pulmonary disease (COPD) with a previous exposure to Asbestos. Moreover, he suffered of cardiac insufficiency and benign prostatic hypertrophy. In June 2015, after an episode of melaena, an esophago-gastro-duodenoscopy (EGDS) found a polypoid formation in the duodenal bulb. Histopathological examination showed epithelioid nests, positive for chromogranin and synaptophysin, with low expression of ki67 (<2%). Considering these features, the neoplasm was recognized as a well-differentiated neuroendocrine neoplasm (Fig. 1). Total body CT scan was performed to define clinical staging. It showed 2 small adrenal gland nodules with 17 and 11 mm of maximum diameter, respectively. They had regular margins and their aspect was consistent with the diagnosis of adenomas. Octreotide scan didn’t show any areas of uptake. In August 2015, the patient started a treatment with intramuscular injections of Octreotide (30 mg every 28 days). In January 2016, there was no significant change at CT scan while 6 months later a new revaluation showed infiltration of dx renal sinus and the third proximal ureter segment with heterogeneous enhancement of vascular structure. An US-guided biopsy of the lesion was performed in August 2016 with the diagnosis of a Diffuse Large B Cell Lymphoma (Figs. 2 and 3). In October 2016, a colonoscopy described a neoplastic lesion at 20 cm from the anal orifice. The Histology exam was positive for an adenocarcinoma, with an infiltrative desmoplastic matrix (Fig. 4). One month later, the patient underwent a left hemicolectomy: the pathologic staging described a G2 adenocarcinoma pT3N1b. One year after the diagnosis of colon cancer, the PET-CT detected several hepatic nodules with a pathologic fluorodeoxyglucose (FDG) intake. The most evident lesion (standardized uptake value [SUV] max = 5.3) was found in the VI segment. In addition, there was a mesenteric nodule metabolically active (SUV 4.8) near surgical clips and a non-specific thickening in the gluteus maximus muscle (SUV = 4.8). Cytofluorometry of blood samples confirmed the persistence of a lymphomatous disease. In May 2018 the Octreotide scan was still negative. During the same month, the patient started a 6 cycles treatment with Rituximab, Oxaliplatin, and Capecitabine. In July 2018, 3 cycles were added. After 6 months, it was decided to continue with FOLFIRI and cetuximab because of progression of liver metastases. The patient is still in treatment.

## 3 Discussion

Neuroendocrine tumors are frequently associated (55% of cases) with other malignancy: synchronous primary tumors (lesions diagnosed 6 months before or after the finding of a NET) or metachronous tumors (second primary tumors identified after 6 months since the discovery of a NET).^[11]^ As Hui-Jen Tsai et al reported, the incidence of a second neoplasm in these patients is 10% to 20%.^[12]^ These tumors are more often detected in the gastrointestinal and genitourinary tracts but they could arise everywhere. In a meta-analysis, the frequency of a second primary tumor in patients affected by NETs was 17% which is 2 times the incidence expected in patients with other non-endocrine tumors.^[11]^ In the Tsai Hui Jen's study, the risk of second primary malignancy after the diagnosis of a neuroendocrine tumor was not significantly different between women and men while the risk of a secondary tumor was related to the age, being higher in patients older than 70 years. The most common cancer recorded were colon cancer and lung cancer independently from the site where the neuroendocrine tumor was located. The risk to develop a second primary tumor was higher in patients with NETs than in general population with a standardized incidence ratios (SIR) of 1.48 (95% CI, 1.09–1.96). The risk to develop bladder and kidney/renal pelvis/urethra cancer was higher with a SIR of 3.68 (95% CI, 1.00–4.93) and 4.48 (95% CI, 1.22–11.48), respectively. The risk was not significantly high for other types of cancers. In a Swedish study, an increased risk of metachronous cancers like small bowel, prostate, skin, endocrine glands cancer and Non-Hodgkin Lymphoma was found in men. In the same study, women had an increased risk of upper airway and digestive tract cancers, small bowel cancer, colon cancer, breast cancer, genitorurinary cancers, melanoma, and leukemia.^[13]^ Several studies showed that second primary tumors occur in different body sites. The prognosis of GEP NETs depends on the location site. NETs of the appendix have good prognosis without relevant symptoms so they are often accidentally diagnosed. Small bowel NETs and traditional carcinoids with a bad prognosis are often metastatic and multicentric when discovered. To define the prognosis properly, histologic, functional and radiologic information are required. Histologic factors include differentiation and proliferation index (Ki67 or mitotic index). The main functional factors to be considered are 18F-FDG PET and Octreotide scan, whereas radiology is important to detect significant differences between 2 subsequent radiologic examinations.^[14]^ The increased risk of a second primary neoplasm after the NET diagnosis might be due to various factors like genetic predisposition, life style, and ongoing treatments. The 5% to 10% of GEP-NENs have a hereditary pattern and they can be found in different syndromes like MEN-1, MEN-2, Von Hippel-Lindau disease, neurofibromatosis, and tuberous sclerosis. Genetic aberrations of MEN1, ATP dependent X-linked helicase (ATRX)/DAXX, mammalian target of rapamycin (mTOR) pathway were also found in the pancreatic sporadic NETs. Genetic instability might increment the potential for the onset of second primary neoplasms. With the aim to explain the association of NETs with other types of cancer, some studies proposed the field-effect theory, which states the existence of a common carcinogenic effect responsible for the onset of both NETs and other tumors.^[11,15]^ NETs produce and release many neuropeptides while SPMs overexpress receptors for these molecules.^[16]^ The above substances have specific properties as growth factors. For instances, Gastrin and cholecystokinin can stimulate the growth of gastric mucosa and pancreatic cells. Gastrin and cholecystokinin are expressed also in lung, ovaries, thyroid, and brain.^[17,18]^ Bombesin stimulates the growth of human breast cancer cells in vitro^[19]^ and it is a powerful growth factor in the small cell lung cancer.^[20]^ In addition, NETs produce other growth factors like platelet-derived growth factor (PDGF), fibroblast growth factor (FGF), transforming growth factor (TGF).^[21]^ A possible therapeutic strategy might be blocking receptors for these neuropeptides. Results of a study by Kamp K et al^[22]^ which involved 459 patients demonstrated that only the occurrence of a synchronous second primary tumor mainly increased in patients with a gastrointestinal neuroendocrine cancer, differently from what previous studies showed. Kauffman RM et al studied a population of 3086 patients with NETs and a second primary cancer. They estimated the incidence of second primary neoplasms in patients with pancreatic NETs and GEP NETs. The conclusion was that patients with gastrointestinal neuroendocrine tumors had a higher risk to develop a second primary neoplasm than those with pancreatic NETs. Interestingly, the last group had also a lower risk than general population.^[23]^ Because there is a remarkable tendency to develop further neoplasms, patients affected by NETs should be always followed using imaging techniques.^[14]^

## 4 Conclusions

Association between NETs and risk to develop a second primary neoplasm is 17%. The most common sites where these malignancies take place are the genitourinary and gastrointestinal tract, independently from where the neuroendocrine neoplasm is located. There is no difference between men and women about the incidence while age is important for the risk assessment because these tumors are more frequent in patients with more than 70 years. However, more studies are required to define other risk and prognostic factors.

## Author contributions

**Conceptualization:** Angelo Pirozzi.

**Data curation:** Angelo Pirozzi, Grazia Arpino, Carmela Mocerino.

**Formal analysis:** Angelo Pirozzi.

**Funding acquisition:** Giacomo Cartenì.

**Investigation:** Angelo Pirozzi.

**Resources:** Severo Campione.

**Supervision:** Ferdinando Riccardi, Carlo Molino.

**Validation:** Grazia Arpino.

**Visualization:** Severo Campione.

**Writing – original draft:** Angelo Pirozzi.

**Writing – review & editing:** Angelo Pirozzi.
